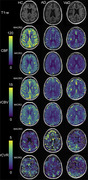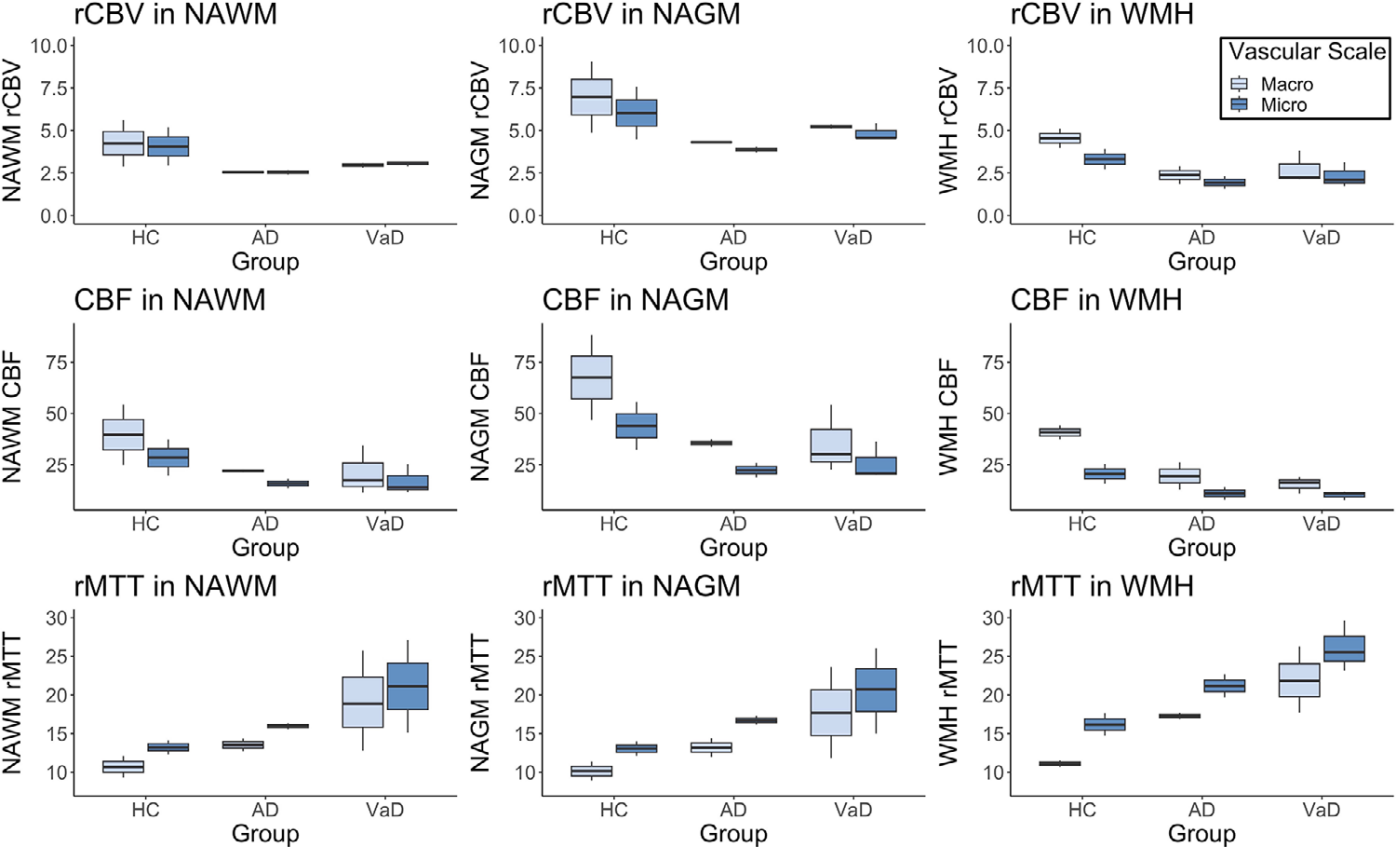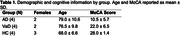# Perfusion and cerebrovascular reactivity characterization in Alzheimer’s disease and vascular dementia

**DOI:** 10.1002/alz.093850

**Published:** 2025-01-09

**Authors:** Elizabeth G. Keeling, Molly M. McElvogue, Lauren R. Ott, Anna D. Burke, Marwan N. Sabbagh, Nadine Bakkar, Ashley M. Stokes

**Affiliations:** ^1^ Barrow Neurological Institute, Phoenix, AZ USA

## Abstract

**Background:**

Cerebrovascular changes are often reported in normal aging, Alzheimer’s disease (AD), and vascular dementia (VaD). Cerebral perfusion and cerebrovascular reactivity (CVR) both decrease with dementia compared to healthy aging; as these changes occur prior to symptomatic onset and in distinct brain regions, perfusion and CVR may act as complementary biomarkers of early cerebrovascular changes. These biomarkers can be measured using MRI methods, yielding macrovascular measures of perfusion and CVR. We recently developed a more advanced method capable of measuring microvascular‐specific measures of perfusion and CVR. Here, we characterized the cerebrovascular profiles of AD and VaD using complementary perfusion and CVR biomarkers representing both macrovascular and microvascular regimes.

**Method:**

MRI data were acquired at 3T (Ingenia, Philips) in three cohorts: non‐cognitively impaired cohort (HC), AD, and VaD (Table 1). Perfusion data were acquired with a multi‐echo, multi‐contrast (SAGE) acquisition (5 echoes, 7.7/26/56/74/92 ms), before, during, and after injection of gadolinium‐based contrast agent. Acquisition parameters include: repetition time (TR) = 1.5 s, voxel size = 2.75×2.75×5 mm, 200 volumes, acquisition time = 5 min. SAGE functional MRI (fMRI) data were acquired in the same cohort with the same TEs and the following acquisition parameters: TR = 3.0 s, voxel size = 3 mm3, 160 volumes, acquisition time = 8 min. SAGE data underwent standard pre‐processing. Macro‐ and microvascular cerebral blood flow (CBF), relative cerebral blood volume (rCBV), and relative CVR (rCVR) were calculated using advanced analysis pipelines. The Montreal Cognitive Assessment (MoCA) was administered prior to MRI acquisition.

**Result:**

As expected, microvascular perfusion and rCVR were lower than the corresponding macrovascular metrics for all groups. Macrovascular and microvascular CBF were lower for AD and VaD compared to HC, while there was no difference in CBF between AD and VaD (Figures 1,2). Similar trends were observed for rCBV. There were no differences in macrovascular rCVR between groups; microvascular rCVR was lower for AD and VaD compared to controls.

**Conclusion:**

Macrovascular and microvascular perfusion decreases with AD and VaD. Enrollment is ongoing, and future directions include analysis of perfusion metrics within cortical and subcortical regions and correlation of neuroimaging findings with cognitive testing.